# Functional or Nonfunctional Cusps Preservation for Molars Restored with Indirect Composite or Glass-Ceramic Onlays: 3D FEA Study

**DOI:** 10.3390/polym13213831

**Published:** 2021-11-05

**Authors:** Pablo Lenin Benitez Sellan, Larissa Mendes Campaner, João Paulo Mendes Tribst, Amanda Maria de Oliveira Dal Piva, Guilherme Schmitt de Andrade, Alexandre Luiz Souto Borges, Eduardo Bresciani, Antonio Lanzotti, Pietro Ausiello

**Affiliations:** 1 School of Dentistry, Universidad Espíritu Santo, Samborondón 092301, Ecuador; pablo.benitez@unesp.br; 2 Institute of Science and Technology, São Paulo State University (Unesp), São José dos Campos, São Paulo 12220-690, Brazil; larissa.m.campaner@unesp.br (L.M.C.); alexander.borges@ict.unesp.br (A.L.S.B.); eduardo.bresciani@unesp.br (E.B.); 3 Department of Dental Materials, Academic Centre for Dentistry Amsterdam (ACTA), University of Amsterdam and Vrije Universiteit Amsterdam, 1081 LA Amsterdam, The Netherlands; joao.tribst@gmail.com (J.P.M.T.); amodalpiva@gmail.com (A.M.d.O.D.P.); 4 Department of Dentistry, Center for Biological and Health Sciences, Western Paraná State University (Unioeste), Cascavel 85819-110, Brazil; guilherme.andrade@unesp.br; 5 Fraunhofer JL IDEAS, Department of Industrial Engineering, University of Naples Federico II, 80125 Naples, Italy; antonio.lanzotti@unina.it; 6 School of Dentistry, University of Naples Federico II, 80131 Naples, Italy

**Keywords:** dental materials, finite element analysis, prosthodontics, biomechanics

## Abstract

Evidence regarding the effect of the onlay preparation design for different CAD/CAM restorative materials considering the preservation of cusps is lacking. Molars were 3D-modeled in four preparation designs for onlay restoration: traditional design with functional cusp coverage (TFC), non-retentive design with functional cusp coverage (NFC), traditional design with non-functional cusp coverage (TNFC) and non-retentive design with non-functional cusp coverage (NNFC). The restorations were simulated with two CAD/CAM restorative materials: LD—lithium disilicate (IPS e.max CAD) and RC—resin composite (GrandioBloc). A 100 N axial load was applied to the occlusal surface, simulating the centric contact point. Von Mises (VM) and maximum principal (Pmax) stress were evaluated for restorations, cement layer and dental substrate. The non-retentive preparation design reduced the stress concentration in the tooth structure in comparison to the conventional retentive design. For LD onlays, the stress distribution on the restoration intaglio surface showed that the preparation design, as well as the prepared cusp, influenced the stress magnitude. The non-retentive preparation design provided better load distribution in both restorative materials and more advantageous for molar structure. The resin composite restoration on thenon-functional cusp is recommended when the functional cusp is preserved in order to associate conservative dentistry and low-stress magnitude.

## 1. Introduction

In the last decade, restorations made using the CAD/CAM methodology have been gaining attention. The lithium disilicate for dental restorations was introduced in 2006 and is marketed in a pre-crystalline phase that, after machining, undergoes a firing process to reach the final selected color [1]. Several clinical studies with full and partial overlay restorations have been carried out with this material, showing promising results [2,3,4]. In a literature review conducted by Pieger et al., including research conducted on lithium disilicate glass-ceramic, a 97.8% survival rate was observed in the five-year follow-up [5].

Although the success rates for dental ceramics are high, there are still some clinical situations that require improvement. There are limitations in posterior teeth, where there is a greater masticatory load than in the anterior teeth, which might compromise posterior restoration, making them more vulnerable to failure [6]. The distribution of occlusal forces in individuals with no occlusal changes is greater in the molar region, reaching an average maximum force of 400 N, while in individuals with some parafunctional habits, as in the cases of bruxism, this force can reach values up to 1000 N [7,8]. Some of the failures that can occur due to the limitations of this type of material are cracks or catastrophic fractures, inadequate marginal adjustment and wear of antagonistic teeth opposed to the restoration [9,10].

The damage tolerance of lithium disilicate does not only depend on the manufacturing method but on the fracture toughness presented by this material as well. The toughness mechanism seems to be influenced by the size of the crystals, since they prevent the spread of cracks that can lead to catastrophic failure [11]. This dental material presents a survival rate of 96.3% in two years for 37 restorations, of which 5 restorations failed and 3 were fractured [12].

A critical factor to consider when comparing the pressed and machined lithium disilicate restorations is the marginal adjustment, which can present mismatch, leading to deleterious effects such as marginal staining, cement degradation or secondary caries [5]. In a review of the two restoration techniques, it was concluded that the presses obtained a smaller marginal crack than the machined ones [13]. In this way, the quality of the preparation for CAD/CAM restorations must be more refined and uniform, with no retentions that can prevent adaptation [14].

Making use of a material that works well with the tooth structure would be an interesting alternative to circumventing the failures of restorations, since commercially there are blocks such as hybrid type ceramics or nanoresins with a greater number of fillers [15,16]. One of these types of blocks was launched in 2013 and is commercially distributed by VOCO under the name of Grandio Blocs. The composition of this material, which includes a 86% inorganic filler content, results in greater strength and stability [17,18].

According to the manufacturer, the elastic modulus of the composite nanoresins shows values between the ceramic and indirect composite resins, thus having a flexural strength of 330MPa [19]. These data led us to believe that this type of material could have a superior clinical behavior in relation to catastrophic failures when compared to porcelain and even glass-ceramics, in addition to reducing the need to wear antagonistic teeth. The indications, according to the manufacturers, are for inlays, onlays, overlays, single crown restorations and in some cases, they can be used in areas of greater stress [20]. 

Cavity preparation design can be one of the main reasons for tooth fracture or restoration [21]. When the tooth loses more of its structure, especially the marginal ridge, the cavity becomes more susceptible to deformations when stressed. Unlike metallic restorations, ceramic restorations with traditional preparations, due to their fragile nature, do not support occlusal loads when they involve the coverage of the cusp of the dental structure [22]. Several studies have offered different designs for the preparation of the cavity, ideal for restorations of partial ceramic covering [23,24,25] as well as for the cement layer polymerization kinetics [25,26].

When the surface of the cavity preparation is flat, even the restoration of the cavity with high adhesive strength material cannot guarantee a stable restoration. The creation of retention can reduce the interfacial adhesive strength and increase the survival rate of the restorations, thus decreasing the probability of early detachment. However, the preparation of the retention grooves sacrifices much of the remaining tooth structure [27]; for this reason, conventional preparations must adopt minimally invasive approaches that avoid restoration failures [28], although preparations for partial restoration lead to considerable enamel loss [22].

With the continuous development of dental materials and ceramics with innovative manufacturing processes such as CAD/CAM, the question that arises is whether the preparation guidelines for partial ceramic restorations could be adapted for minimally invasive approaches while preserving more healthy tissue. Therefore, the aim of the present study was to biomechanically analyze various preparation designs for lithium disilicate ceramic and resin composite onlays under simulated tensions induced by chewing. In particular, we aimed to determine if the risk of fracture is greater with lithium disilicate ceramic restorations than with CAD/CAM resin restorations.

## 2. Materials and Methods

A three-dimensional geometry was obtained with the aid of CAD (Computer Aided Design) software (Rhinoceros^®^ version 4.0; McNeel North America, Seattle, WA, USA). The 3D teeth were obtained from a scan of preparations made in resin models, in which the teeth presented anatomy based on the literature [15]. In this step, the models were generated from lines drawn over the image in STL, in which the main anatomical landmarks are chosen, with lines generated over them that referenced the surface. Molars were modeled in 4 preparation designs of onlay restoration: traditional with functional cusp coverage (TFC), non-retentive with functional cusp coverage (NFC), traditional with non-functional cusp coverage (TNFC) and non-retentive with non-functional cusp coverage (NNFC). 

The design for the traditional onlay preparation was created with a 2.7 mm depth, 2.3 mm opening of the isthmus and a 1.2 mm opening of the gingival wall. The cavity walls of the preparation were tapered from 6 to 10°. The functional (2 mm) and non-functional (1.5 mm) cusps were reduced considering the specific design of each group. The non-retentive preparation following the natural tooth morphology (2 mm on functional cups, 1.5 mm on non-functional cusp), all angles and walls smooth and rounded [23,24,25,26,27].

**Figure 1:**
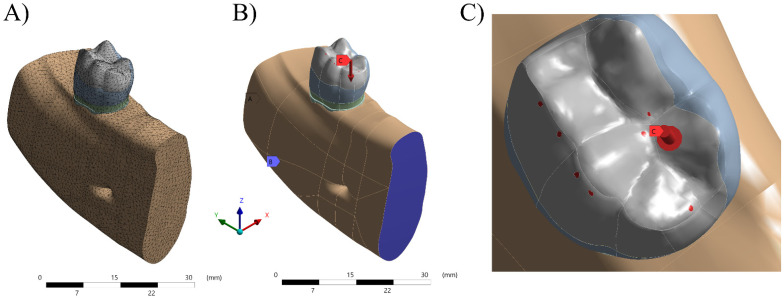
Numerical model processing. (**A**) Mesh generation. (**B**) Fixation of the system. (**C**) Axial load application in occlusal points.

After forming the surfaces, they were transformed into solids to provide delimitation of the structures that were studied. These volumes were exported in STP format (Standard for the Exchange of Product Model Data) so that the software (ANSYS 17.2, ANSYS Inc., Houston, TX, USA) for pre- and post-processing of finite element analysis could be used. The designed structures were tooth with onlay preparation (functional or non-functional cusp), onlay, resin cement layer, periodontal ligament and alveolar bone composed by cortical and cancellous bone tissues (Figure 1). 

After importing the geometries, the meshes were made, and the density was adjusted to obtain sufficient and accurate results. For that, the Ansys 19.2 Software (ANSYS Inc., Houston, TX, USA) was used, in which meshes of quadratic tetrahedral elements are used, characterized by pyramids with a triangular base, with a knot in each vertex and another in the center of each edge, totaling 10 nodes per element. This type of volume element is the most suitable for reproducing complex and curved geometries such as dental structures, as it adapts better spatially, thus being a very powerful tool for representing volumes of anatomical geometries.

After editing the models, their specific properties were assigned. At this stage, it is important to check the consistency of the physical quantities used in the model together with the homogeneity of metric systems. In this study, the proper analysis to represent the fracture test was performed by structural static analysis, in which at least w properties, among them the elastic modulus (E) in GPa and Poisson’s ratio (V), must be informed. Thus, each geometry has specific properties that characterized its behavior for an assay within the limits of linearity (Table 1) [18,28,29,30,31,32].

The relationship between the geometries determines the transmission of the existing tensions from one element to other at the interface region; thus, it is necessary to define the contacts. In this study, they were all considered bonded.

For the simulation of the boundary conditions in all models, the displacement in all directions was restricted and the load was applied by simulating a force of 100 N [33]. A vertical load on the occlusal surface perpendicular to the load axis simulating the centric contact point was applied.

## 3. Results

The visualization of the results of analysis by finite element method was performed qualitatively using a color stress map, in which each fringe represents a range of stress or deformations generated in the evaluated structures. For the analysis of the results, the maximum principal stress (MPa) was used to assess the stress distribution and magnitude in the restoration, cement layer and dental substrate.

**Figure 2:**
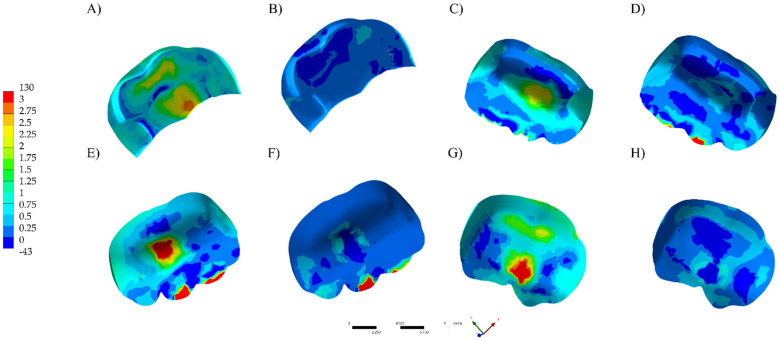
Maximum principal stress distribution in the restoration’s intaglio surface. (**A**) LD TFC. (**B**) RC TFC. (**C**) LD TNFC. (**D**) RC TNFC. (**E**) LD NFC. (**F**) RC NFC. (**G**) LD NNFC. (**H**) RC NNFC.

**Figure 3:**
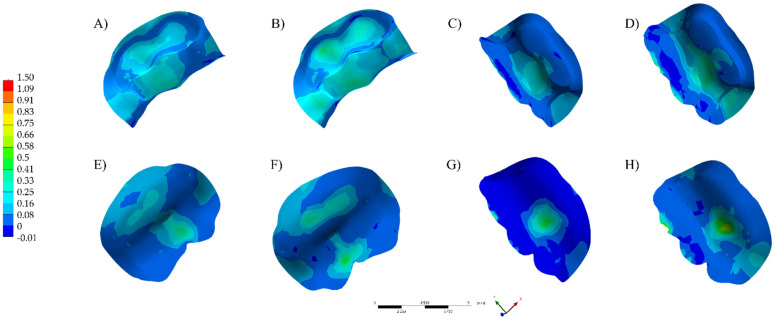
Maximum principal stress distribution in the cement layer. (**A**) LD TFC. (**B**) RC TFC. (**C**) LD TNFC. (**D**) RC TNFC. (**E**) LD NFC. (**F**) RC NFC. (**G**) LD NNFC. (**H**) RC NNFC.

**Figure 4:**
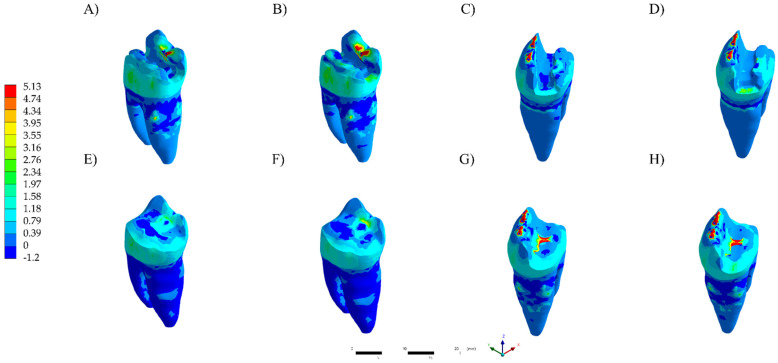
Maximum principal stress distribution in the tooth structure. (**A**) LD TFC. (**B**) RC TFC. (**C**) LD TNFC. (**D**) RC TNFC. (**E**) LD NFC. (**F**) RC NFC. (**G**) LD NNFC. (**H**) RC NNFC.

The numerical results are plotted in colorimetric stress maps in Figure 2, Figure 3 and Figure 4, and the highest values of the Tensile Stress (MPa) are summarized in Table 2.

The stress on the tooth structure (enamel and dentin) was calculated using the Maximum probe detected by the Mechanical APDL (ANSYS 19.2, ANSYS Inc., Houston, TX, USA). Observing the stress distribution on the restoration’s intaglio surface (Figure 2), LD onlays (130–95 MPa) showed higher tensile stress concentration in the center of restoration (Figure 2A,C,E,G) than RC. For RC models, the stress peaks were higher (130 MPa) when TFC preparation design was considered. For LD onlays, the stress distribution on the restoration’s intaglio surface showed that the preparation design influenced the stress distribution as well as the prepared cusp. Regardless of the restorative material, the non-retentive preparation design improved the distribution of the stress concentration to the tooth structure in comparison to the conventional retentive design. However, for conventional preparation, the stress concentration was higher than the restoration itself.

## 4. Discussion

The traditional retentive onlay preparation protocols and guidelines were based on classic studies [34,35], in which healthy tissue was often not preserved and cusp coverage was needed if the cavity extension was two-thirds or greater than the distance from any primary groove to the tip of the cusp. Based on the results of the finite element analysis of this study, the non-retentive preparation design showed a more favorable behavior and reduced the concentration of stress on the tooth structure compared to the conventional retentive design.

Restorative dentistry has currently experienced a dramatic increase in minimally invasive restorations. Dentists, in order to preserve as much dental structure as possible, are constantly changing the traditional guidelines for the design of non-retentive preparations [23]. In addition, scientific evidence on enamel and dentin adhesion techniques are reliable, and studies have shown adequate mechanical behavior for non-retentive preparations under load [36]. These data corroborate the present study, considering that the non-retentive preparation presented the lowest stress concentration values in all the analyzed structures. The less retentive preparation usually has a greater amount of enamel, and the tissue has better adhesion.

Dental adhesive systems play a vital role in today’s restorative dentistry practices [37]. These adhesive restorations substantially decrease the amount of tooth reduction required during the cavity preparation process, since adhesive restorations do not require extensions for retention [22,23,24,25]. It is important to note that most of the margin of the restoration is placed on the enamel, which serves as an excellent bonding surface for the adhesive system. Therefore, the present study’s results suggest that the retention longevity and functionality of the restoration can therefore be increased.

The decision to cover the functional or non-functional cusp in partial coverage preparations may be due to the amount of remaining dental structure [24,38]. In the present study, the reduction of functional or non-functional cusps was compared; the results demonstrated that when the non-functional cusp was prepared, the stress on the functional cusp increased regardless of the type of preparation. From a clinical point of view, when restoring teeth that have already loose the non-functional cusp, a non-retentive preparation associated with composite resin can be an alternative treatment in order to be more conservative.

The use of ceramics is needed in dental treatments where the traditional form of mechanical retention is limited and may allow non-retentive dental preparations, as shown in the stress distribution values on the internal surface of the restoration in this study. As the technologies and CAD/CAM materials have been improved and are being further developed, the design of the preparation margin will not present a significant difference during the manufacturing process [39,40].

As no consensus has been reached on how much to wear in onlay preparations today, the guidelines should be geared towards making decisions about how to prepare and what type of material is most appropriate. In line with this clinical philosophy, resin and ceramic onlays are now considered viable alternatives to full-coverage crowns, with a success rate exceeding 90% in 10 years [41]. However, manufacturers fail to provide guidance on when cusp coverage with ceramic material is needed.

The design of this study focused on covering the functional or non-functional cusp with two types of preparation of the lower first molar tooth; the results are applicable only for this type of case. The designs of non-retentive preparations used in this study can, theoretically, minimize the concentration of stress on the tooth structure; however, this is transferred to the restoration, so it is important to follow those restorations while in service.

## 5. Conclusions

Despite the limitations of this study, which considers only the stress magnitude, it is possible to conclude that:

1. For the first molar rehabilitation, both restorative materials present a suitable applicability during onlay treatments; however, preferable non-retentive preparation designs should be performed.
2. The resin composite restoration on non-functional cusp is recommended when functional cusp is preserved in order to associate conservative dentistry and low stress magnitude.

## Figures and Tables

**Table 1:** Materials properties considered in the present study.

Material	Elastic Modulus (GPa)	Poisson Ratio	Reference
Enamel	84.1	0.33	[28]
Dentin	18.6	0.32	[28]
Lithium Disilicate (e.max CAD)	102.7	0.21	[29]
Resin Composite (Grandio Blocs)	18	0.26	[18]
Resin cement (Multilink N)	8.3	0.7	[18,30]
Periodontal Ligament	0.050	0.49	[31]
Cortical Bone	12.6	0.25	[32]
Cancellous bone	1.14	0.32	[32]

**Table 2:** Stress peaks (MPa) measured for each geometry according to the preparation and restorative material combination.

Material	Design	Tooth	Cement Layer	Onlay
Lithium Disilicate	Traditional	32.3	0.6	130.1
		43.1	0.8	95.9
	Non-retentive	25.1	0.6	103.6
		41.1	0.5	109.1
Resin Composite	Traditional	36.2	1.2	130.4
		42.1	1.6	95.5
	Non-retentive	26.1	1.1	105.2
		38.3	0.6	103.4

## Data Availability

Data available on request.
